# Longitudinal changes of serum immunoglobulins under ocrelizumab: factors associated with hypogammaglobulinemia and infection risk in a real-world multiple sclerosis cohort

**DOI:** 10.1186/s42466-026-00509-0

**Published:** 2026-06-29

**Authors:** Amelie Bohn, Fiona S. Teubner, Simone C. Tauber, Anne Waschbisch

**Affiliations:** Department of Neurology, RWTH Aachen University, Aachen, Germany

**Keywords:** Multiple Sclerosis, B-cell depletion, Immunoglobulins, Hypogammaglobulinemia, Infection Risk

## Abstract

**Background and Objectives:**

Ocrelizumab (OCR) is an effective anti-CD20 therapy for relapsing-remitting and primary progressive multiple sclerosis (MS), but long-term effects on humoral immunity in real-world settings remain incompletely characterized. We investigated longitudinal changes in serum immunoglobulins, explored factors associated with hypogammaglobulinemia, and assessed associations with infection risk.

**Methods:**

In this retrospective, single-center observational study, adult patients with MS treated with OCR between 2018 and 2023 were included after ≥ 2 treatment cycles. Patients underwent standardized clinical, radiological, and laboratory follow-up, including serial measurements of serum immunoglobulins and varicella-zoster virus (VZV) serology. Infections were graded according to CTCAE criteria.

**Results:**

Of 116 patients (81% relapsing-remitting MS; median follow-up 2.3 years), approximately one-third developed lower limit of normal (LLN) IgM and one-sixth LLN IgG. Declines in immunoglobulins were class-specific and treatment-duration-dependent, most pronounced for IgM. Low baseline immunoglobulin levels were the strongest factors associated with LLN values, whereas demographic and disease-related factors were not. Prior immunotherapy, particularly fingolimod or interferon, was associated with hypogammaglobulinemia. Severe infections were rare (1.1 per 100 patient-years) and not consistently associated with LLN immunoglobulins.

**Discussion:**

OCR induced predictable, class-specific declines in immunoglobulins. Despite frequent hypogammaglobulinemia - particularly affecting IgM - no association with an increased short- to mid-term risk of clinically relevant infections was observed, although limited event numbers restrict conclusions. These findings support individualized immunoglobulin monitoring focused on patients with low baseline levels or prior immunotherapy exposure and underscore the need for prospective studies to define clinically meaningful thresholds for intervention during long-term anti-CD20 therapy.

**Supplementary Information:**

The online version contains supplementary material available at 10.1186/s42466-026-00509-0.

## Introduction

Over the past decade, B-cell-depleting monoclonal antibodies have transformed the therapeutic landscape for relapsing and progressive forms of Multiple Sclerosis (MS). Ocrelizumab (OCR), a humanized anti-CD20 antibody, has demonstrated robust efficacy in reducing inflammatory disease activity and slowing disability progression in both relapsing-remitting MS (RRMS) and primary progressive MS (PPMS) [1, 2]. Its widespread adoption in clinical practice has therefore been accompanied by growing interest in understanding its long-term immunological effects in real-world settings.

OCR induces rapid and sustained peripheral B-cell depletion, a mechanism closely linked to its clinical benefits but also potential downstream consequences for humoral immunity [3]. Immunoglobulin (Ig) levels can decline over time during anti-CD20 therapy [4]. While reductions in IgM are common and often occur early, decreases in IgG are slower and more moderate [4, 5]. Observational studies have suggested that cumulative exposure to OCR or prior disease-modifying therapy (DMT) may increase the risk of hypogammaglobulinemia, a complication for which clear guidelines on subsequent management are lacking, yet findings have been inconsistent and limited by controlled study conditions, heterogeneous monitoring practices, and lack of detailed longitudinal characterization [5–8]. A key clinical question is whether treatment-related Ig decline translates into an increased risk of infections. Although OCR has been associated with mild-to-moderate infectious complications, the relationship between quantitative Ig trajectories, patient-level risk factors, and infection outcomes remains incompletely understood [4, 7, 9–11]. Furthermore, herpes zoster has emerged as a specific concern with several immunosuppressive MS therapies; however, data on its incidence and serologic correlates under OCR remain sparse, particularly outside controlled trial settings [1, 12, 13].

Real-world cohorts provide an opportunity to characterize the variability and dynamics of Ig changes under routine care, identify factors associated with hypogammaglobulinemia (HG), and determine whether declines in humoral immunity are associated with an increased infection burden. Until now, questions remain about the appropriate monitoring intervals, the threshold values that warrant intervention, and the management strategies for patients who develop clinically relevant HG [6].

In this study, we investigated longitudinal changes in serum IgG, IgM, and IgA in a well-characterized real-world cohort of OCR-treated patients with MS. We aimed to (1) quantify class-specific patterns of Ig decline across treatment cycles, (2) identify demographic, clinical, and treatment-related factors associated with the development of Ig levels below the lower limit of normal (LLN), and (3) evaluate the association between Ig dynamics and the occurrence of infections, including herpes zoster. By integrating multi-year laboratory monitoring with detailed clinical phenotyping, this work provides a comprehensive assessment of humoral immune changes during OCR therapy and their potential relevance for long-term safety management in MS treatment.

## Material & methods

### Study population

This retrospective, single-center observational study was conducted at the Department of Neurology, University Hospital RWTH Aachen, a tertiary care center specializing in MS management. The following inclusion criteria were applied: (1) age ≥ 18 years, (2) diagnosis of RRMS or PPMS according to the McDonald criteria valid at that time [14], and (3) OCR treatment between February 1, 2018, and April 30, 2023. Exclusion criteria included: (1) prior therapy with other B-cell-depleting agents, (2) OCR initiation outside the study center, (3) fewer than two OCR treatment cycles, and (4) fewer than two available Ig measurements.

### Setting

OCR therapy was administered within the Department of Neurology at University Hospital RWTH Aachen. Prior to the first infusion, each patient underwent a comprehensive baseline assessment by a neurologist, including neurological examination, EDSS scoring, and estimation of walking distance based on patient history. Brain (± spinal) MRI scans were performed annually, typically in an outpatient setting. Vaccination status was reviewed and, if indicated, updated according to the recommendations of the Robert Koch-Institute.

In the absence of clinical or laboratory evidence of active or chronic infection, OCR infusions were prepared and dispensed by the hospital’s pharmacy. The first cycle consisted of two 300 mg doses given two weeks apart, followed by subsequent 600 mg cycles every six months (singular (*n* = 10) infusion intervals were delayed during the coronavirus pandemic). Administration followed the manufacturer’s protocol. Premedication consisted of intravenous dimenhydrinate (4 mg), intravenous dexamethasone (12 mg), and oral paracetamol (1 g). Following OCR treatment initiation, follow-up visits with the referring outpatient neurologist were scheduled every 2–6 months and consisted of clinical history and neurological examination. Laboratory testing was typically performed about two weeks before each scheduled OCR infusion.

### Data collection

This retrospective study was conducted in accordance with the principles of the Declaration of Helsinki and approved by the institutional ethics committee of the University Hospital RWTH Aachen (approval number: 23–181, date: 10.07.2023). Due to the retrospective nature of the study and the use of anonymized data, the requirement for informed consent was waived by the ethics committee. The routinely collected health data were retrieved retrospectively from *Medico*, the hospital’s electronic health record system. Collected variables included sociodemographic characteristics (age, sex, height, weight) and disease-related parameters (comorbidities, MS subtype, disease duration, EDSS score, MRI-detected disease activity, reported infections during treatment, infusion-related reactions (IRRs), hospitalizations, number of OCR cycles, intervals between cycles, prior DMTs, symptomatic medications, high-dose corticosteroid treatment, and plasmapheresis). Laboratory data (assessed approximately every 2–6 months) consisted of a complete blood count, lymphocyte subset immunophenotyping, serum IgG, IgM, and IgA levels at baseline and follow-up, liver enzymes, C-reactive protein (CRP), and serology for chronic infections (HIV, hepatitis B/C), as well as VZV serology. Patients with missing baseline Ig values were retained in longitudinal analyses if follow-up measurements were available, except where explicitly stated otherwise for specific analyses. Urinalysis was performed prior to each infusion to screen for urinary tract infection (UTI), and in women of childbearing potential, pregnancy testing was conducted. MS disease duration was calculated as the difference between age at diagnosis and age at first OCR infusion, expressed in years. Current age was defined as the age at the last OCR infusion or at treatment discontinuation. Height and weight were recorded by patient history.

Measures of MS disease activity included MRI scans and EDSS scoring. MRI-based disease activity was defined as new or enlarging T2 lesions or gadolinium-enhancing T1 lesions. EDSS was reassessed at least annually, typically before each OCR infusion, with walking distance estimated by patient report. Prior to each OCR administration, patients were systematically assessed for infections that had occurred since the previous infusion during routine clinical visits. For reported infections, information on therapeutic interventions was obtained, and available external medical records were reviewed, including outpatient and primary care reports. Infection severity was retrospectively graded according to the Common Terminology Criteria for Adverse Events (CTCAE, v5.0) for infections, based on documented clinical information, including the need for antimicrobial therapy and hospitalization [15]. Recurrent infections in the same patient were recorded as separate events if they were clinically distinct and temporally separated. Lymphopenia severity was defined as grade 1 (< 1000 to ≥ 800 cells/µL), grade 2 (500–799 cells/µL), grade 3 (200–499 cells/µL), and grade 4 (< 200 cells/µL). Neutropenia severity was defined as grade 1 (< 1600 − 1500 cells/µL), grade 2 (< 1500 − 1000 cells/µL), grade 3 (< 1000 − 500 cells/µL), and grade 4 (< 500 cells/µL) [15]. Reference ranges for normal Ig levels were 700–1500 mg/dL for IgG, 40–250 mg/dL for IgM, and 70–450 mg/dL for IgA. Values below these ranges were classified as hypogammaglobulinemia, with IgG graded as follows: grade 1, 400–700 mg/dL; grade 2, 200–399 mg/dL; grade 3, 0-200 mg/dL. Anti-VZV IgM and IgA were considered positive if the IgM index was ≥ 1.0 and the IgA ratio was ≥ 1.0 according to the reference values of our in-house laboratory.

### Statistical analysis

Data were analyzed using GraphPad Prism 10 (GraphPad Software Inc., San Diego, CA) and SPSS version 29.02 (IBM Corp., Armonk, NY, USA). The normality of distribution was evaluated using the Shapiro-Wilk test. Nominal and ordinal data were compared using Fisher’s exact test or generalized estimating equations (GEE) for repeated measures. In the presence of complete separation in 2 × 2 contingency tables, effect size estimation was performed using Firth’s penalized logistic regression to reduce small-sample bias and to obtain finite, reliable parameter estimates. Comparisons were performed between baseline Ig levels and each subsequent treatment cycle, as well as between consecutive cycles. IgG levels were analyzed as normally distributed metric data, while IgM and IgA values were logarithmically transformed to approximate normality. These variables were evaluated using a repeated-measures mixed-effects model followed by Šídák’s multiple comparisons test. Anti-VZV-IgG/IgM/IgA and total Ig levels, stratified by baseline quartiles, were analyzed using a repeated-measures mixed-effects model with Tukey’s post hoc multiple comparisons test. For comparisons within Ig quartiles, normally distributed variables were analyzed using Brown-Forsythe ANOVA, whereas non-normally distributed variables were analyzed using the Kruskal-Wallis test. For subgroup analyses, baseline EDSS score was dichotomized at ≥ 3.5 to represent moderate disability. Additional variables, including age, disease duration, BMI, prior DMT use, and number of OCR cycles, were dichotomized at the cohort median. In univariable analyses, age was further categorized as ≥ 40 versus < 40 years, based on the median rounded to the nearest whole year. Nominal variables are presented as counts and percentages, while ordinal and continuous variables are reported as medians with interquartile ranges (IQRs). All statistical tests were two-tailed, with significance thresholds defined as *p ≤ 0.05, **p ≤ 0.01, and ***p ≤ 0.001.

## Results

### Study cohort and clinical characteristics

#### Study population

**Fig. 1:**
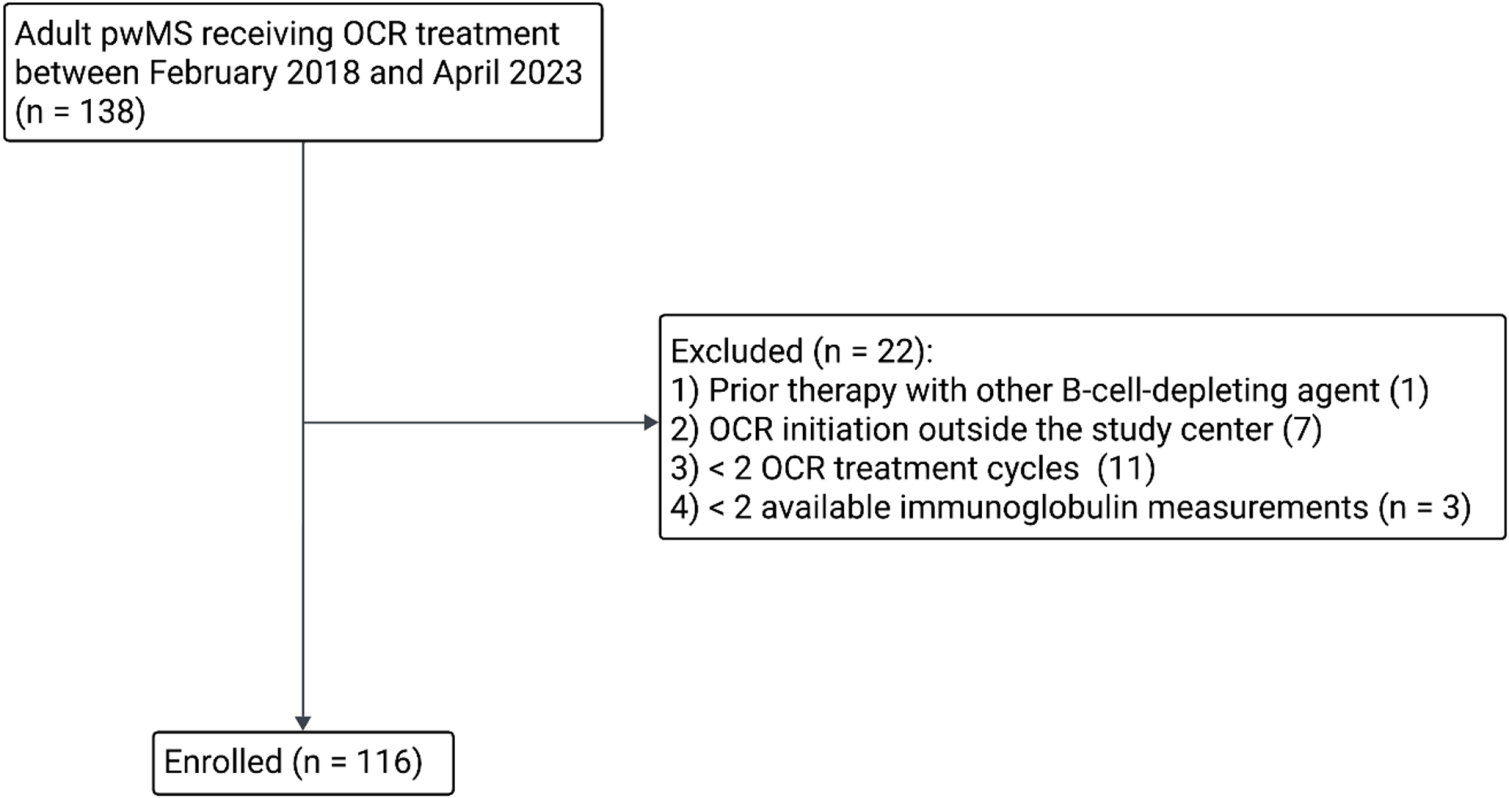
Patient Selection. OCR = Ocrelizumab; pwMS = patients with Multiple Sclerosis

We screened 138 adult patients with MS (pwMS, RRMS or PPMS) who received OCR treatment between February 2018 and April 2023. Twenty-two patients were excluded according to the predefined exclusion criteria (Fig. 1), resulting in a final analysis cohort of 116 patients. This cohort included 9 patients who discontinued OCR treatment due to either severe COVID-19-associated acute respiratory distress syndrome (*n* = 1), disease activity (*n* = 1), loss to follow-up (*n* = 4), pregnancy (*n* = 1), patient decision (*n* = 1), or comorbidity requiring inpatient alcohol withdrawal treatment (*n* = 1).

#### Baseline characteristics

Demographic and clinical characteristics of the study cohort are summarized in Table 1. Patients were predominantly female (60%) and diagnosed with RRMS (81%). The median age at MS onset was 28 years (IQR 23–41), and OCR treatment was initiated at a median age of 39 years (IQR 30–52), corresponding to a median disease duration of 8 years (IQR 4–12). The median EDSS at treatment start was 2.5 (IQR 1.0-4.5). Comorbidities were common, most frequently psychiatric disorders (47%). Malignancies were rare (3%) and limited to isolated cases of non-small cell lung cancer, malignant melanoma, and cervical dysplasia. Patients in this cohort had notable prior treatment, with a median of two previous DMTs (IQR 1–3), most commonly interferon beta (59%), glatiramer acetate (44%), fingolimod (40%), and natalizumab (40%). During a median follow-up of 2.3 years (IQR 1–4), patients received a median of five OCR cycles (IQR 2–6).

**Table 1:** Demographics and clinical characteristics

Variable	
Clinical/Demographic factors	
Female sex, n (%)	70 (60)
Median body mass index, kg/m² (IQR) (*n* = 71)	25 (22–30)
RRMS, n (%)	94 (81)
Median age at MS onset, years (IQR)	28 (23–41)
Median age at MS diagnosis, years (IQR)	29 (23–42)
Median age at first OCR treatment, years (IQR)	39 (30–52)
Median MS disease duration at OCR treatment initiation, years (IQR)	8 (4–12)
Median EDSS at OCR treatment initiation, score (IQR) (*n* = 105)	2.5 (1-4.5)
Median number of OCR cycles (IQR)	5 (2–6)
Median follow-up duration, years (IQR)	2 (1–4)
Discontinuation of OCR treatment, n (%)	9 (8)
Patients with LLN IgG (< 700 mg/dL) at baseline (*n* = 107), n (%)	12 (11)
Patients with LLN IgG (< 700 mg/dL) during OCR treatment, n (%)	18 (16)
Patients with LLN IgM (< 40 mg/dL) at baseline (*n* = 107), n (%)	11 (10)
Patients with LLN IgM (< 40 mg/dL) during OCR treatment, n (%)	43 (37)
Patients with LLN IgA (< 70 mg/dL) at baseline (*n* = 107), n (%)	8 (8)
Patients with LLN IgA (< 70 mg/dL) during OCR treatment, n (%)	9 (8)
Comorbidities, n (%)	
Psychiatric	55 (47)
Others (e.g., hypothyroidism)	23 (20)
Cardiovascular	15 (13)
Autoimmune	11 (10)
Neurological	11 (10)
Malignant disease	3 (3)
Number of DMT-naive patients, n (%)	8 (9)
Number of pwRRMS (n = 94) per prior DMT, n (%)	
Interferon beta-1a and beta-1b	55 (59)
Glatiramer acetate	41 (44)
Fingolimod	38 (40)
Natalizumab	38 (40)
Dimethyl fumarate	28 (30)
Teriflunomide	4 (3)
Cladribine	2 (2)
Alemtuzumab	1 (1)
Median number of prior DMTs in pwRRMS (*n* = 94), n (IQR)	2 (1–3)
MRI-detected disease activity per OCR treatment year, n (%)	
at OCR treatment initiation (*n* = 109)	65 (60)
in the 1st year (*n* = 94)	19 (20)
in the 2nd year (*n* = 72)	4 (6)
in the 3rd year (*n* = 51)	5 (10)
in the 4th year (*n* = 33)	1 (3)
in the 5th year (*n* = 17)	1 (6)
pwRRMS (*n* = 92) with EDSS progression (increase of ≥ 1 point) during OCR, n (%)	25 (29)
Median EDSS in pwRRMS per OCR treatment year, score (IQR)	
at OCR treatment initiation (*n* = 87)	2.0 (1.0-4.5)
in the 1st year (*n* = 87)	2.0 (1.0-4.5)
in the 2nd year (*n* = 76)	2.0 (1.0-4.5)
in the 3rd year (*n* = 61)	2.0 (1.0-4.5)
in the 4th year (*n* = 36)	3.0 (1.0-5.5)
in the 5th year (*n* = 24)	3.5 (1.5-6.0)
Median EDSS in pwPPMS per OCR treatment year, score (IQR)	
at OCR treatment initiation (*n* = 18)	4.5 (3.5-6.0)
in the 1st year (*n* = 18)	5.0 (4.0–6.0)
in the 2nd year (*n* = 18)	5.5 (4.5-6.0)
in the 3rd year (*n* = 15)	6.0 (5.0-6.5)
in the 4th year (*n* = 11)	6.0 (5.0-6.5)
in the 5th year (*n* = 4)	6.5 (3.5–6.5)
Median number of infusion-related reaction per cycle, n (%)	4 (7)

DMT = disease-modifying therapy; EDSS = Expanded Disability Severity Scale; LLN = lower limit of normal; MRI = magnetic resonance imaging; MS = Multiple Sclerosis; OCR = ocrelizumab; pwPPMS = patients with primary progressive Multiple Sclerosis; pwRRMS = patients with relapsing-remitting Multiple Sclerosis

For calculations in which data were not available from all patients, the corresponding *n* denotes the number of individuals included in that specific analysis. All results, except for the EDSS, are rounded to whole numbers. MRI-based disease activity was defined as new or enlarging T2 lesions or gadolinium-enhancing T1 lesions. EDSS was reassessed at least annually, typically before each OCR infusion, with walking distance estimated by patient report

#### Outcomes

OCR initiation was followed by a marked decline in MRI-based disease activity, consistent with a significant annual reduction (OR 0.29, 95% CI 0.19–0.44, *p* < 0.001). Before treatment, 60% of patients showed active MRI lesions, decreasing to 20% in the first year and ≤ 10% in subsequent years. Disability progression (EDSS increase ≥ 1 point) occurred in approximately one-third of patients with relapsing-remitting MS (pwRRMS) and those with primary progressive MS (pwPPMS). However, GEE showed no significant association between EDSS progression and increasing treatment years in either pwRRMS (OR 1.31, 95% CI 0.95–1.82, *p* = 0.10) or pwPPMS (OR 1.27, 95% CI 0.80-2.00, *p* = 0.33). Among pwRRMS, 27% required high-dose corticosteroids for relapses during OCR treatment. Use was highest shortly after initiation and decreased thereafter to ≤ 8% (Supplementary Table 1), resulting in a significant decrease in corticosteroid use with advancing OCR cycles (OR 0.79, 95% CI 0.66–0.94, *p* = 0.008).

#### Infusion-related reactions

Infusion-related reactions occurred in 7% of OCR infusion cycles, peaking at cycle 1 (18%) and declining thereafter (Supplementary Table 1), resulting in a significant cycle-dependent reduction in IRR risk (OR 0.87, 95% CI 0.79–0.97; *p* = 0.013).

### Laboratory monitoring during ocrelizumab treatment

Regular monitoring of the blood count during OCR treatment is essential to ensure both efficacy and safety. Hematological values across treatment cycles are summarized in Supplementary Tables 2 A-B. Median routine blood count parameters remained within the reference range throughout treatment. Median neutrophil and lymphocyte counts were also stable, though 27 patients experienced lymphopenia, and 4 patients experienced neutropenia at least once during treatment, all of which were limited to grade 1 severity.

### Longitudinal changes in immunoglobulin levels during OCR treatment

Baseline Ig levels showed substantial interindividual variability. Of the 116 patients included in the cohort, baseline Ig values were available for 108 patients. One patient was excluded from the analysis due to erroneous blood sampling during ongoing plasma exchange, which resulted in markedly low pre-treatment Ig values.

IgG declined gradually, with modest reductions through cycles 1–4 and a more pronounced decrease from cycles 5–6, reaching − 17.9% at cycles 9–10. IgM declined earlier and more markedly, peaking at -50.5% in cycles 7–8. IgA declined moderately, reaching − 31.0% at cycles 9–10, with an overall pattern less pronounced than IgM and comparable to IgG.

**Fig. 2:**
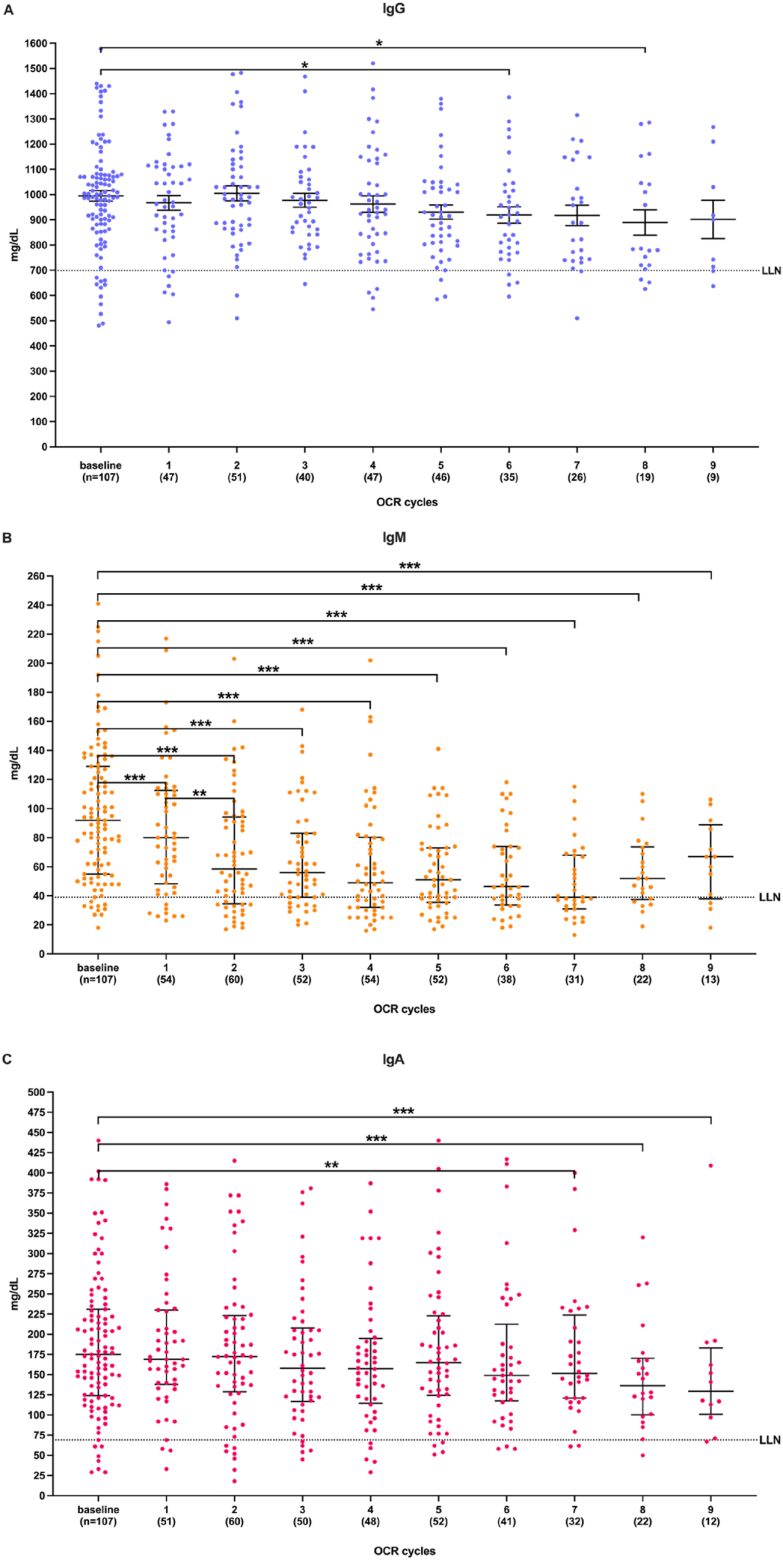
Mean Serum Immunoglobulin Levels during Ocrelizumab Treatment. X-axis: baseline and OCR cycles 1–9; y-axis: IgG (**A**), IgM (**B**), IgA (**C**) in mg/dL. Data is shown as mean ± SEM (**A**) or median ± IQR (**B**-**C**); dashed lines indicate the LLN/ Grade 1 hypogammaglobulinemia threshold. *n* denotes the number of values included in each calculation. Patients with missing baseline immunoglobulin levels were included in the analysis provided that subsequent immunoglobulin measurements were available. IgM and IgA were log-transformed (Y = log(Y)) for statistical modeling. Analyses were performed using REML with Šídák multiple comparisons. p values were set at *p ≤ 0.05, **p ≤ 0.01, and ***p ≤ 0.001. Ig = immunoglobulin; LLN = lower limit of normal; OCR = ocrelizumab

**Fig. 3:**
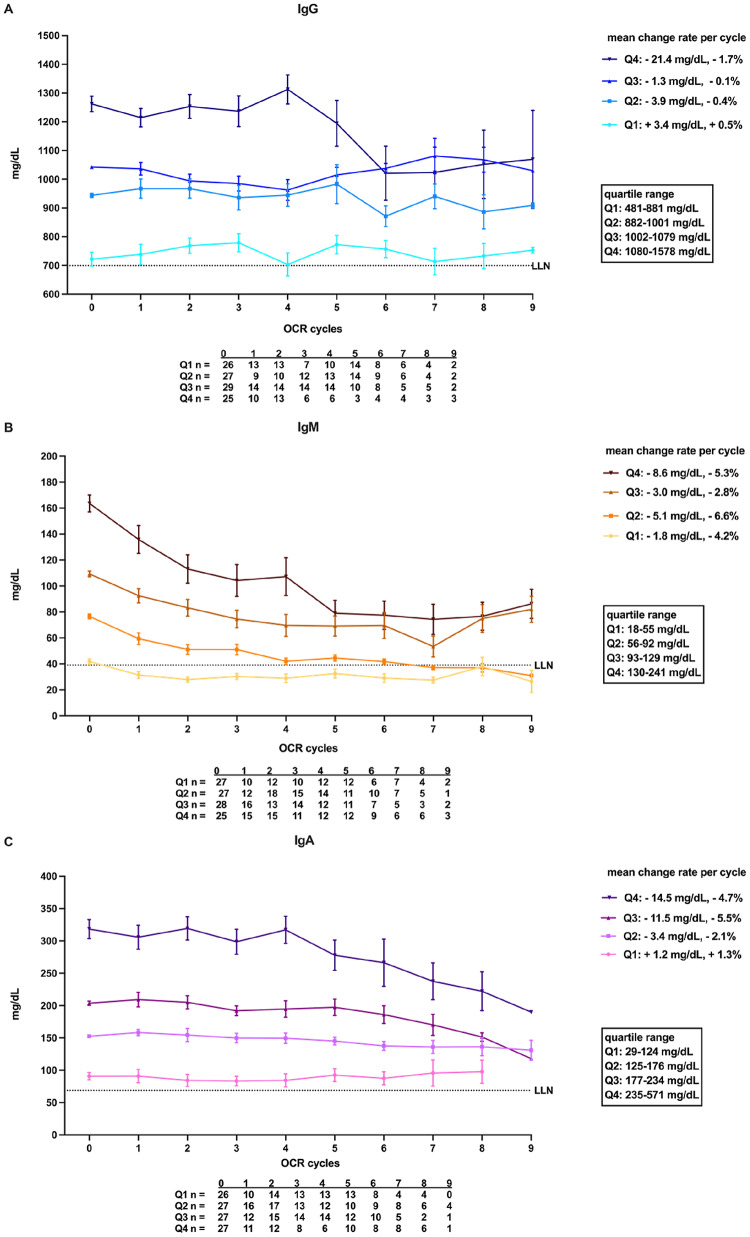
Longitudinal Changes of Immunoglobulin Levels by Baseline Quartiles during Ocrelizumab Treatment The x-axis shows baseline (0) and OCR cycles 1–9, the y-axis displays serum immunoglobulin concentrations (mg/dL). Data are presented as mean ± SEM, with the lower limit of normal (LLN) indicated by dashed lines (IgG: 700 mg/dL; IgM: 40 mg/dL; IgA: 70 mg/dL). n denotes the number of values contributing to each mean, which decreases in later cycles due to staggered treatment initiation. For Q1 IgA levels from cycle 9 were not available. Patients with missing baseline immunoglobulin levels were excluded from these analyses, as stratification was performed based on baseline immunoglobulin values. The mean rate of change per cycle was calculated by subtracting each subsequent value from the preceding one and averaging these differences across cycles. Statistical analysis was performed using a REML, and within-quartile changes were evaluated using Tukey’s multiple comparisons test. *p *values were set at *p ≤ 0.05, **p ≤ 0.01, and ***p ≤ 0.001. OCR = ocrelizumab. **A**) IgG levels remained stable across quartiles, and Tukey’s test estimated no significant reductions vs. baseline. (Effect sizes and 95% CIs as reported in Supplementary Table 3). **B**) Significant IgM declines were observed within all quartiles. Quartile 1: Significant reductions in cycles 1-4 and 7. Quartile 2: Significant reductions in cycles 1–8. Quartile 3: Significant reductions in cycles 1-7. Quartile 4: Significant reductions in cycles 2–8. (Effect sizes and 95% CIs as reported in the Supplementary Table 4). **C**) IgA showed significant within-quartile reductions beginning at cycle 5. Q1 lacked cycle 9 data. Quartiles 1-4: Significant reductions occurred in cycles 5-8 (Q1) and cycles 5-9 (Q2-Q4). (Effect sizes and 95% CIs as reported in the Supplementary Table 5)

To characterize these trends in greater detail, absolute Ig levels are presented in Fig. 2. Figure 2A displays mean ± SEM IgG levels, while Figs. 3B-C show median IgM and IgA levels with IQR due to non-Gaussian distributions. Cycle 10 data were omitted because only two measurements were available.

Baseline IgG ranged from 481 to 1578 mg/dL, with 11.2% below the LLN. IgG declined by 1.0% (10.2 mg/dL) per cycle. Across cycles, 1–8% of patients had grade 1 HG, and 15.5% experienced grade 1 at least once (vs. 11.2% at baseline). No grade 2 or 3 HG occurred. REML with Šídák correction showed significant IgG reductions at cycles 6–8 (*p* ≤ 0.05), but mean values never fell below the LLN (700 mg/dL).

Baseline IgM ranged from 18 to 241 mg/dL, with 10.3% below the LLN. IgM declined by 6.1% (5.9 mg/dL) per cycle. The proportion of patients with LLN IgM varied from 7 to 30%, and 37% experienced LLN IgM at least once (increase of 26.4% from baseline). IgM decreased significantly at every cycle compared with baseline and from cycle 1 to 2 (*p* ≤ 0.01), with the steepest drop during the first two cycles. Median IgM crossed the LLN threshold (40 mg/dL) at cycle 7.

Baseline IgA ranged from 29 to 440 mg/dL, with 7.5% below the LLN. IgA declined by 1.7% (3.2 mg/dL) per cycle. The proportion of patients with LLN IgA remained low (2.5-6.0%), and 7.8% showed LLN IgA at least once (0.3% increase vs. baseline). Significant IgA reductions occurred at cycles 7–9 (*p* ≤ 0.01), but mean levels remained above the LLN (70 mg/dL) throughout treatment. 

Fig. 3 illustrates longitudinal changes in serum IgG, IgM, and IgA stratified by baseline quartiles. Across all three Ig classes, individuals in higher baseline quartiles consistently exhibited higher absolute concentrations throughout treatment, yet all groups demonstrated a progressive decline over time. At low baseline Ig levels, there was no disproportionate decline over time compared with patients who had high baseline Ig levels. Although patients in the lowest quartile remained closest to the LLN, mean IgG and IgA values in all quartiles remained above the threshold throughout treatment. However, for IgM, patients in the lower two baseline quartiles were more likely to reach the LLN during follow-up

### Factors associated with hypogammaglobulinemia

**Fig. 4:**
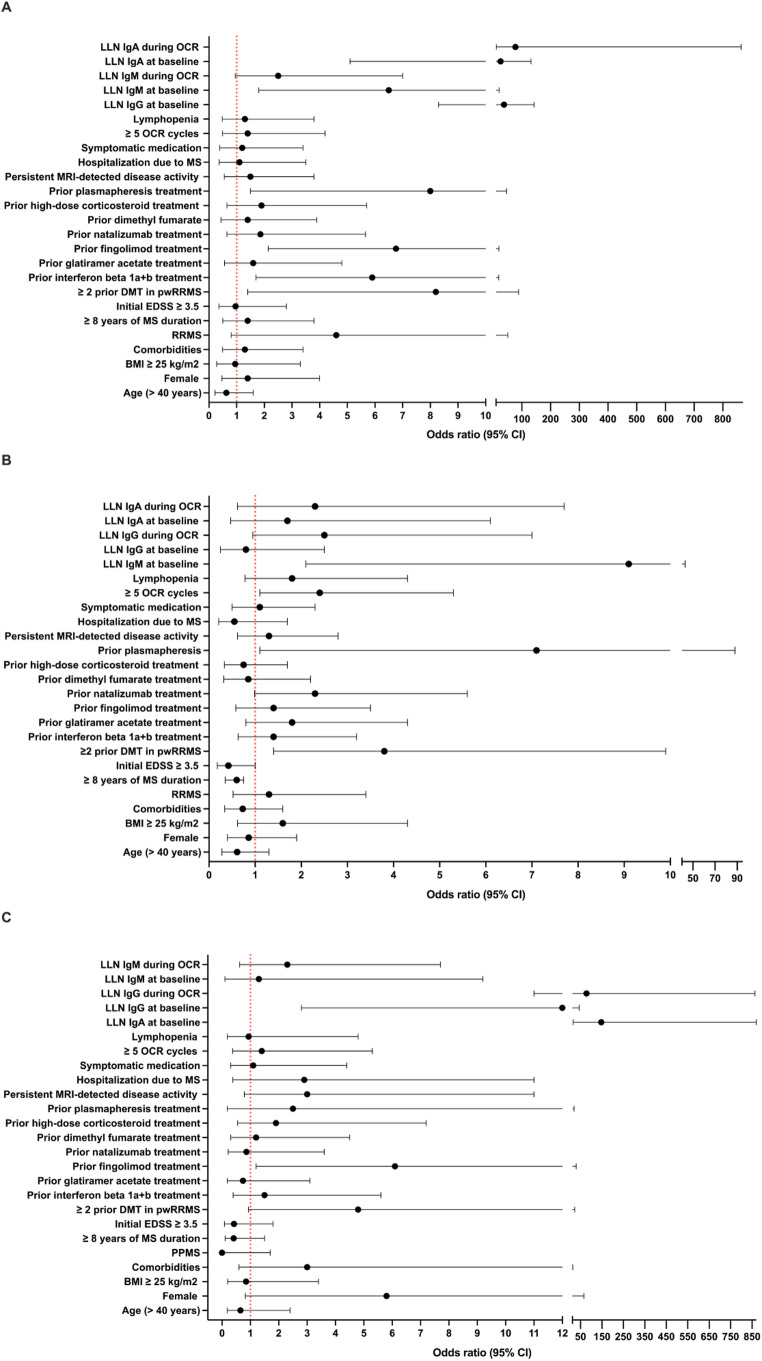
Univariable Analysis of Clinical and Treatment-Related Factors Associated with LLN Immunoglobulin Levels. Univariable analysis evaluated clinical and treatment-related factors associated with immunoglobulin levels falling below the LLN for IgG (**A**), IgM (**B**), and IgA (**C**) in the study cohort (*n* = 116). Each point represents the odds ratio (OR) with 95% confidence intervals. ORs > 1 indicate increased odds of LLN immunoglobulin levels (IgG < 700 mg/dL, IgM < 40 mg/dL, IgA < 70 mg/dL) occurring at least once during treatment. Each dot represents the odds ratio, with horizontal lines indicating the 95% CIs. Due to incomplete data, sample sizes varied for the following variables: BMI *n* = 71, EDSS *n* = 105, LLN IgG at baseline *n* = 107, LLN IgM at baseline *n* = 107, LLN IgA at baseline *n* = 107. Analyses of prior DMTs were performed exclusively in patients with relapsing-remitting Multiple Sclerosis. Univariable analyses were performed using Fisher’s exact test. DMT = disease-modifying therapy; EDSS = Expanded Disability Severity Scale; Ig = immunoglobulin; LLN = lower limit of normal; MS = Multiple Sclerosis; OCR = ocrelizumab; PPMS = primary progressive Multiple Sclerosis; RRMS = relapsing-remitting Multiple Sclerosis

Following confirmation of significant declines in IgG, IgM, and IgA during OCR therapy, univariable analyses were performed to identify potential clinical and demographic factors associated with the occurrence of hypogammaglobulinemia (Figs. 4A-C).

For IgG, LLN IgG was significantly associated with ≥ 2 prior DMTs (OR 8.20, 95% CI 1.40–89.00, *p* = 0.02), prior plasmapheresis (time interval between OCR initiation and plasmpharesis < 1.3 years, median 75 days; OR 8.00, 95% CI 1.50–47.00, *p* = 0.04), prior interferon beta-1a/1b (OR 5.90, 95% CI 1.70–20.00, *p* = 0.006), and prior fingolimod treatment (OR 6.76, 95% CI 2.14–20.09, *p* = 0.001). Prior interferon therapy in the absence of combined fingolimod exposure was also associated with LLN IgG (OR 11.96, 95% CI 1.19-∞, *p* = 0.04). Baseline LLN IgG showed a strong association with on-treatment LLN IgG (OR 38.0, 95% CI 8.30–143.0, *p* < 0.01). Additional associated factors included low baseline IgM (OR 6.50, 95% CI 1.80–21.0, *p* = 0.01) and low baseline or on-treatment IgA (baseline: OR 26.0, 95% CI 5.10–132.0; during treatment: OR 78.0, 95% CI 11.0-864.0; both *p* < 0.01).

For IgM, ≥ 2 prior DMTs (OR 8.20, 95% CI 1.40–89.00, *p* = 0.02) and the receipt of ≥ 5 OCR cycles (OR 2.40, 95% CI 1.10–5.30, *p* = 0.05) were associated with increased odds of developing IgM levels below the LLN. Low baseline IgM showed a strong association with on-treatment LLN IgM (OR 9.10, 95% CI 2.10–43.0, *p* < 0.01). No other clinical variables reached significance.

For IgA, prior fingolimod exposure (OR 6.10, 95% CI 1.20–30.00, *p* = 0.03) and concurrent LLN IgG at baseline (OR 12.0, 95% CI 2.80–44.0, *p* < 0.01) as well as during treatment (OR 78.0, 95% CI 11.0-864.0, *p* < 0.01) were associated with LLN IgA. Baseline LLN IgA showed the strongest association (OR 147.0, 95% CI 16.0-870.0, *p* < 0.01).

### Infections

During follow-up, 34 patients (29.3%) reported at least one infection, corresponding to an overall rate of 16.1 per 100 patient-years. Urinary tract infections (UTIs) were the most common, affecting 19 patients (16.4%), including 17 uncomplicated (14.7%) and 2 complicated cases (1.7%). Respiratory tract infections (RTIs) occurred in 12 patients (10.3%), comprising 8 common colds (6.9%), 3 sinusitis episodes (2.6%), and one case of otitis media (0.9%). Additional infections included herpes zoster (4.3%), cutaneous mycosis (1.7%), and gastroenteritis (1.7%). COVID-19 cases (*n* = 41) were not included in the primary infection analysis due to their distinct epidemiological context during the pandemic, including highly fluctuating infection rates, public health measures, and vaccination rollout, which may differ from other infections and potentially confound longitudinal safety assessments.

Most infections were mild, with a median CTCAE grade of 1 (IQR 1–2). Severe infections (≥ CTCAE grade III) occurred in 3 male patients (2.6%), corresponding to an incidence of 1.1 severe infections per 100 patient-years. These included two complicated UTIs (cycles 2 and 6) and one case of appendicitis with septicemia (cycle 6). One had LLN IgG at baseline and during treatment, and another showed LLN IgM during treatment.

Univariable analysis comparing patients with and without infections identified no significant associations with most demographic, disease-related, or treatment-related factors (Supplementary Fig. 1). Receiving ≥ 5 OCR cycles was associated with a higher probability of documented infections (OR 4.70, 95% CI 1.80–12.00, *p* < 0.01) in Fisher’s exact test. However, this variable is inherently linked to longer follow-up duration and therefore reflects increased opportunity for event detection. Consistently, the infection rate per treatment cycle did not increase with longer cumulative infusion duration (Supplementary Table 1). Surprisingly, high-dose corticosteroid treatment was associated with fewer documented infections (OR 0.35, 95% CI 0.14–0.95, *p* = 0.04).

To further assess the impact of excluding COVID-19 cases, a sensitivity analysis was performed re-evaluating the association between LLN Ig levels and infection occurrence, including COVID-19-related infections. This analysis confirmed the absence of an association and showed that results remained unchanged when COVID-19 cases were included (Supplementary Table 7).

### Herpes zoster and anti-VZV antibody responses

Baseline VZV immune status was available for all patients (100%), all of whom demonstrated sufficient VZV-specific immunity based on serological evidence, consistent with prior infection or vaccination. Five patients developed clinically confirmed herpes zoster during OCR treatment and were compared with those without herpes zoster (*n* = 111). Univariable analysis (Supplementary Table 8, Supplementary Fig. 2) revealed no significant associations between herpes zoster occurrence and demographic, disease-related, or treatment-related factors. Anti-VZV IgG, IgM, and IgA levels were assessed annually to monitor humoral responses and identify potential serologic signs of VZV reactivation. Due to staggered treatment initiation, the number of available measurements decreased in later cycles. Despite considerable interindividual variability, no overall trend toward increasing anti-VZV titers (IgG), indices (IgM), or ratios (IgA) was observed (Supplementary Fig. 3). Median anti-VZV IgG concentrations remained stable and consistently above the positivity threshold (≥ 100 mIU/mL). Median anti-VZV IgM indices and anti-VZV IgA ratios remained below the cutoff for positivity (≥ 1.0). Among the five patients with herpes zoster, anti-VZV antibody results were available concurrently with the clinical episodes for only two of the five patients with herpes zoster. Both showed increases in anti-VZV IgA ratios to ≥ 1.0 (0.8→1.3 and 0.1→2.0).

## Discussion

In this real-world longitudinal cohort of OCR-treated patients with MS, we provide a detailed characterization of class-specific Ig trajectories, explore factors associated with hypogammaglobulinemia, and examine their clinical relevance with respect to infection risk. Several key findings emerge. First, OCR was associated with a consistent, treatment-duration-dependent decline in serum Ig levels, following a reproducible hierarchy with the earliest and most pronounced reduction in IgM, a more gradual and moderate decline in IgG, and comparatively minor changes in IgA. Second, baseline Ig concentrations were the strongest and most consistent factors associated with the occurrence of levels below the LLN during treatment, outweighing demographic or disease-related variables. Third, despite measurable declines in humoral immunity, documented clinically relevant infections were infrequent, predominantly mild, and not associated with on-treatment hypogammaglobulinemia. Together, these findings support the notion that Ig decline under OCR is common but heterogeneous, closely related to baseline values, and of limited short- to mid-term clinical consequence in most patients. However, this interpretation is constrained by the low number of severe infection events.

The observed Ig patterns align closely with prior clinical trial and observational data [1, 11, 16]. Consistent with pivotal trial extensions and post-marketing studies, IgM exhibited an early and steep decline, frequently crossing the LLN after several treatment cycles, whereas IgG declined slowly and generally remained within the reference range at the cohort level. The relative preservation of IgA observed here further supports the concept that long-lived plasma cells, which lack CD20 expression, remain largely unaffected by OCR, while memory B-cell depletion preferentially impacts IgM-producing compartments [17]. Importantly, by leveraging dense longitudinal sampling under routine care, our data extend prior work by illustrating substantial interindividual variability and demonstrating that absolute Ig levels largely track with baseline quartiles throughout treatment. This finding underscores that OCR induces parallel downward shifts rather than convergence toward a uniform nadir, a clinically relevant distinction when interpreting laboratory results.

A central contribution of this study is the identification of factors associated with hypogammaglobulinemia. Across all Ig classes, low baseline levels emerged as the dominant associated factor for subsequent LLN values during therapy, a finding that is consistent with observations from other real-world studies [7, 18]. Prior treatment exposure was associated with an increased risk of hypogammaglobulinemia, with multiple prior DMTs linked to low LLN IgG and IgM, prior fingolimod exposure to low LLN IgG and IgA, prior interferon beta exposure to low LLN IgG, and prior plasmapheresis to LLN IgG and IgM, likely reflecting pre-existing immune compromise.

Importantly, interferon beta has not previously been highlighted as a factor associated with hypogammaglobulinemia following subsequent B-cell depletion. Most available studies evaluating prior treatment effects under anti-CD20 therapy have analyzed interferon in combination with other low- to moderate-efficacy agents [8, 19], thereby limiting attribution to a specific compound. Mechanistically, interferon beta therapy has been shown to reduce the immature/transitional B-cell pool while increasing plasmablast frequencies and upregulating BAFF expression, thereby altering B-cell subset homeostasis [20, 21]. Although not classically immunosuppressive, these shifts may reshape the architecture of the B-cell compartment and could potentially reduce its regenerative flexibility. Subsequent CD20-mediated depletion may be associated with a more pronounced decline in IgG due to a diminished capacity for B-cell reconstitution and long-term maintenance of antibody-producing populations. However, these interpretations remain speculative. In contrast, the association between prior fingolimod exposure and hypogammaglobulinemia is biologically more intuitive. Fingolimod induces sequestration of lymphocytes within secondary lymphoid organs via functional antagonism of S1P receptors, resulting in a marked reduction of circulating B-cell subsets - particularly memory B cells - and has been associated with reduced Ig levels in B-cell-depleting therapy [8, 22, 23]. Initiation of anti-CD20 therapy in this setting effectively eliminates an already numerically and functionally constrained B-cell pool. The combined effect may be associated with a more pronounced hypogammaglobulinemia by further impairing the regenerative capacity of the humoral immune compartment.

These results are consistent with earlier reports suggesting that cumulative immunomodulatory burden predisposes patients to Ig decline under anti-CD20 therapy [8, 24], but our findings emphasize that baseline laboratory assessment provides a simple and powerful tool for clinical stratification. In contrast, age, sex, weight, disease duration, disability status, and MS subtype were not consistently associated with hypogammaglobulinemia, suggesting that routine demographic factors have limited utility in identifying humoral immune changes under OCR [7].

Despite clear laboratory evidence of declining Ig levels, the clinical relevance with respect to infections appeared limited in this cohort. The overall infection rate was comparable to those reported in real-world studies, with UTIs and RTIs predominating and the vast majority of events being mild [7, 9, 11, 25]. Notably, overall infection rates in our cohort were lower than those reported in phase III, pharmaceutical industry-controlled clinical trials [4], most likely reflecting underreporting of mild infections in real-world settings. In contrast, rates of severe infections were largely comparable, as these events are more reliably captured outside of trials due to the need for antibiotic treatment, hospitalization, or other intensive medical interventions. Severe infections were rare and occurred in a small number of patients without a consistent Ig profile. Although an integrated safety analysis of phase III trials reported higher serious infection rates among patients with IgG below the LLN compared with those above it [4], we did not observe a robust association between LLN IgG or IgM and infection risk. However, given the limited number of events, a clinically meaningful association cannot be excluded. A sensitivity analysis including COVID-19-related infections - given their substantial number during the study period - confirmed that no association between LLN Ig levels and documented infections was observed. This finding is consistent with several observational cohorts that failed to demonstrate a clear quantitative threshold at which declining Igs translate into clinically meaningful susceptibility [7, 9, 10, 26]. While receiving ≥ 5 cycles of OCR was associated with an increased overall risk of infection, the infection rate per treatment cycle did not increase with longer cumulative exposure. This indicates that although the probability of experiencing at least one infection naturally rises over time due to longer observation and cumulative treatment, prolonged OCR therapy does not appear to elevate the frequency of infections on a per-cycle basis. The finding that high-dose corticosteroid treatment was associated with fewer infections was unexpected and should be interpreted with caution. This association likely reflects confounding by indication or surveillance bias, as patients receiving corticosteroids for relapses are often younger and may have lower baseline disability and comorbidity burden.

Herpes zoster occurred infrequently and without identifiable associated clinical or laboratory factors, consistent with prior reports indicating a slightly increased but overall low risk under OCR [1, 2]. In contrast to a previously published case report describing loss of VZV-vaccine-induced immunity after the first OCR infusion, anti-VZV IgG levels in our cohort remained stable over time, suggesting preservation of long-term humoral immunity against VZV despite B-cell depletion [27]. The observed transient increases in anti-VZV IgA in two patients with clinically manifest herpes zoster may point toward a mucosal or early humoral response to viral reactivation; however, elevated IgA ratios were also detected in patients without clinically apparent herpes zoster, rendering the significance of possible subclinical reactivation uncertain. Importantly, in these clinically asymptomatic patients, no antiviral treatment was initiated, and no apparent adverse consequences were observed. Overall, our data do not support routine anti-VZV serologic monitoring beyond standard vaccination assessment, although vigilance for clinical symptoms remains warranted.

While neutropenia was infrequent and limited to mild cases in our cohort, isolated reports of recurrent late-onset neutropenia under anti-CD20 therapies such as ofatumumab indicate that rare hematologic complications may still occur during long-term treatment despite otherwise favorable safety profiles [28].

Several limitations merit consideration. The retrospective, single-center design limits causal inference and generalizability, and the modest sample size particularly in later treatment cycles reduces power to detect rare events such as severe infections or profound hypogammaglobulinemia. Some variables analyzed in this study, including the number of OCR cycles and on-treatment Ig levels, are inherently time-dependent and therefore susceptible to exposure-time bias. Patients with longer follow-up have a higher probability of both developing LLN Ig levels and experiencing infections. Infection ascertainment relied on clinical documentation and may underestimate mild events managed outside the hospital system. While VZV serostatus was available for all patients, detailed information on prior varicella or herpes zoster vaccination was not systematically captured due to the retrospective study design. Additionally, Ig measurements reflect serum concentrations and do not capture functional antibody responses or cellular immune competence. Moreover, as recently emphasized by Elgenidy et al. [29], the absence of a standardized definition of hypogammaglobulinemia represents an important limitation in the field and hampers direct comparison of reported rates across studies. In addition, the relatively high proportion of corticosteroid pulse therapies administered during OCR treatment should be interpreted with caution. A relevant share of these pulses was given in the outpatient setting and may not necessarily reflect confirmed relapse activity; therefore, corticosteroid administration alone should not be considered a reliable surrogate marker of true inflammatory disease activity in this cohort.

In conclusion, this real-world longitudinal study demonstrates that OCR induces predictable, class-specific declines in serum Ig levels, and that the occurrence of Ig levels below the LLN is primarily associated with low baseline levels and prior disease-modifying treatment exposure, rather than patient demographics or disease characteristics. While hypogammaglobulinemia - particularly of IgM - is common, no clear association with short- to mid-term infection risk was observed, although the study was not powered to exclude such an effect. These findings support regular but individualized Ig monitoring, with particular attention to patients with low baseline levels or prior immunotherapy. In the context of evolving concepts regarding treatment de-escalation and discontinuation in MS, longitudinal immune profiling may help refine individualized risk-benefit assessments during prolonged anti-CD20 therapy [30]. Future prospective studies integrating functional immune assays and longer follow-up will be essential to define clinically meaningful thresholds and inform evidence-based management strategies for hypogammaglobulinemia under long-term anti-CD20 therapy.

## Supplementary Information

Below is the link to the electronic supplementary material.

Supplementary Material 1

## Data Availability

Anonymized data not published within this article due to reasons of sensitivity will be made available by request from any qualified investigator. Data are located in controlled access data storage at the Department of Neurology, University Hospital RWTH Aachen.

## Abbreviations

CRP: C-reactive protein
CTCAE: Common terminology criteria for adverse events
DMT: Disease-modifying therapy
EDSS: Expanded disability severity scale
GEE: Generalized estimating equations
HG: Hypogammaglobulinemia
Ig: Immunoglobulin
IQR: Interquartile range
IRRs: Infusion-related reactions
LLN: Lower limit of normal
MRI: Magnetic resonance imaging
OCR: Ccrelizumab
PPMS: Primary progressive multiple sclerosis
pwMS: Patients with Multiple Sclerosis
pwPPMS: Patients with primary progressive multiple sclerosis
pwRRMS: Patients with relapsing-remitting multiple sclerosis
RRMS: Relapsing-remitting multiple sclerosis
RTI: Respiratory tract infection
UTI: Urinary tract infection
