# Supplementary material for: Longitudinal changes of serum immunoglobulins under ocrelizumab: factors associated with hypogammaglobulinemia and infection risk in a real-world multiple sclerosis cohort
Source: Neurol Res Pract. 2026 Jun 29;8(1):52. doi: 10.1186/s42466-026-00509-0 (PMC13317294; doi:10.1186/s42466-026-00509-0)
Supplement: Supplementary file 1 — Supplementary Material 1 [file 42466_2026_509_MOESM1_ESM.docx]

**Supplementary Data:**

Longitudinal Changes of Serum Immunoglobulins Under Ocrelizumab: Factors Associated with Hypogammaglobulinemia, and Infection Risk in a Real-World Multiple Sclerosis Cohort

*sTable 1: Prior DMT Exposure, Symptomatic and Acute Treatments, Infusion Reactions, and Infections per Ocrelizumab Cycle in Patients with Multiple Sclerosis*

| **Variable** |  |
| --- | --- |
| **Number of prior DMTs in pwRRMS (n = 94), n (%)** |  |
| 0 | 8 (9) |
| 1 | 19 (20) |
| 2 | 32 (34) |
| 3 | 19 (20) |
| 4 | 13 (14) |
| 5 | 3 (3) |
| **Median number of symptomatic treatments during OCR (IQR)** | 1 (0-2) |
| **Symptomatic treatment, n (%)** |  |
| Antidepressants | 34 (29) |
| Antispasmodics | 21 (18) |
| Fampridine | 20 (17) |
| NSAIDs | 19 (16) |
| Medications for neuropathic pain | 19 (16) |
| Metamizole | 3 (3) |
| **Number of patients with prior plasmapheresis, n (%)** | 5 (4) |
| **High-dose corticosteroid treatment due to MS relapse per cycle, n (%)** |  |
| at OCR treatment initiation (n = 94) | 7 (7) |
| 1 (n = 94) | 9 (10) |
| 2 (n = 90) | 7 (8) |
| 3 (n = 84) | 3 (4) |
| 4 (n = 67) | 3 (5) |
| 5 (n = 57) | 1 (2) |
| 6 (n = 44) | 3 (7) |
| 7 (n = 34) | 2 (6) |
| 8 (n = 30) | 3 (10) |
| 9 (n = 24) | 1 (4) |
| 10 (n = 12) | 0 (0) |
| **Infusion-related reactions per cycle, n (%)** |  |
| 1 (n = 116) | 21 (18) |
| 2 (n = 112) | 11 (10) |
| 3 (n = 99) | 11 (11) |
| 4 (n = 80) | 5 (6) |
| 5 (n = 69) | 3 (4) |
| 6 (n = 55) | 5 (9) |
| 7 (n = 43) | 1 (2) |
| 8 (n = 36) | 1 (3) |
| 9 (n = 28) | 3 (11) |
| 10 (n = 12) | 1 (8) |
| **Number of infections per patient per cycle of OCR, n (%)** |  |
| 1 (n = 116) | 7 (6) |
| 2 (n = 112) | 8 (7) |
| 3 (n = 99) | 7 (7) |
| 4 (n = 80) | 8 (10) |
| 5 (n = 69) | 6 (9) |
| 6 (n = 55) | 7 (12) |
| 7 (n = 43) | 1 (2) |
| 8 (n = 36) | 3 (8) |
| 9 (n = 28) | 0 (0) |
| 10 (n = 12) | 0 (0) |
| Abbreviations: DMT = disease-modifying therapy; MS = Multiple Sclerosis; NSAIDs = non-steroidal anti-inflammatory drugs; OCR = ocrelizumab; pwRRMS = patients with relapsing-remitting Multiple Sclerosis.  For calculations in which data were not available from all patients, the corresponding *n* denotes the number of individuals included in that specific analysis. | |

*sTable 2A: Hematological Laboratory Values across Ocrelizuamb Treatment Cycles*

| **Cycle** | **WBC/nL**  **4.00-10.00** | **RBC/nL**  **3.90-5.20** | **Hb g/dL**  **11.70-15.70** | **PLT/nL**  **150.00-400.00** | **NEUT/nL**  **1.60-7.10** | **NEUT %**  **34.00-71.00** | **LYMPH/nL**  **1.00-2.90** | **LYMPH %**  **19.00-52.00** |
| --- | --- | --- | --- | --- | --- | --- | --- | --- |
| 0 | 6.60  (5.20-7.70)  (n = 105) | 4.80  (4.46-5.20)  (n = 105) | 14.20  (13.10-15.20)  (n = 106) | 251.00  (214.50-295.00)  (n = 105) | 3.67  (2.82-4.52)  (n = 54) | 61.10  (51.93-67.68)  (n = 96) | 1.71  (1.15-2.36)  (n = 54) | 27.90  (21.00-35.90)  (n = 99) |
| 1 | 6.30  (5.15-7.53)  (n = 101) | 4.90  (4.59-5.20)  (n = 102) | 14.35  (13.48-15.40)  (n = 102) | 267.00  (227.25-308.75)  (n = 100) | 3.94  (3.04-4.76)  (n = 58) | 61.70  (56.45-67.35)  (n = 93) | 1.47  (1.13-1.89)  (n = 58) | 24.40  (19.95-29.25)  (n = 93) |
| 2 | 6.45  (5.40-7.68)  (n = 88) | 4.90  (4.60-5.20)  (n = 90) | 14.60  (13.50-15.58)  (n = 89) | 261.00  (234.25-304.00)  (n = 88) | 3.90  (3.14-4.41)  (n = 51) | 63.10  (57.33-67.08)  (n = 88) | 1.46  (1.08-1.90)  (n = 51) | 24.05  (20.85-29.00)  (n = 88) |
| 3 | 6.20  (5.25-7.50)  (n = 73) | 4.90  (4.53-5.20)  (n = 72) | 14.40  (13.50-15.58)  (n = 72) | 265.00  (231.00-295.00)  (n = 71) | 3.65  (3.16-4.77)  (n = 45) | 61.10  (56.55-64.98)  (n = 68) | 1.50  (1.17-1.92)  (n = 45) | 24.60  (21.55-29.55)  (n = 69) |
| 4 | 6.75  (5.68-7.63)  (n = 62) | 4.80  (4.55-5.20)  (n = 61) | 14.20  (13.25-15.30)  (n = 61) | 275.00  (237.00-310.00)  (n = 60) | 3.95  (2.89-4.84)  (n = 48) | 61.40  (55.25-67.65)  (n = 61) | 1.63  (1.33-1.94)  (n = 49) | 25.50  (19.00-31.60)  (n = 61) |
| 5 | 6.30  (5.49-7.58) (n = 56) | 4.90  (4.60-5.20)  (n = 56) | 15.00  (14.13-15.58)  (n = 56) | 275.00  (239.00-297.00)  (n = 57) | 3.68  (3.14-4.50)  (n = 43) | 60.00  (53.00-65.00)  (n = 53) | 1.66  (1.35-2.28)  (n = 43) | 25.60  (21.90-32.50)  (n = 53) |
| 6 | 6.55  (5.35-8.28)  (n = 46) | 4.88  (4.50-5.35)  (n = 46) | 15.15  (14.18-15.75)  (n = 46) | 274.50  (237.25-308.00)  (n = 46) | 3.90  (3.30-5.35)  (n = 39) | 62.45  (57.90-67.30)  (n = 42) | 1.60  (1.34-2.02)  (n = 39) | 24.15  (19.23-30.10)  (n = 42) |
| 7 | 6.50  (5.40-7.80)  (n = 35) | 4.97  (4.60-5.30)  (n = 35) | 15.20  (14.20-16.00)  (n = 35) | 280.00  (252.00-327.00)  (n = 35) | 3.46  (2.86-5.02)  (n = 26) | 60.70  (53.25-64.48)  (n = 32) | 1.80  (1.18-2.08)  (n = 26) | 24.65  (22.23-30.30)  (n = 32) |
| 8 | 6.70  (5.20-8.40)  (n = 27) | 4.80  (4.59-5.30)  (n = 27) | 14.70  (14.10-15.80)  (n = 27) | 292.50  (246.00-318.50)  (n = 26) | 3.99  (3.08-4.95)  (n = 23) | 59.60  (54.45-65.45)  (n = 25) | 1.71  (1.31-2.53)  (n = 23) | 26.00  (20.75-31.45)  (n = 25) |
| 9 | 6.55  (5.63-8.00)  (n = 12) | 4.88  (4.53-5.42)  (n = 12) | 15.55  (13.70-15.90)  (n = 12) | 292.00  (264.00-320.00)  (n = 11) | 3.93  (3.02-4.60)  (n = 9) | 63.90  (52.50-67.20)  (n = 11) | 1.69  (1.15-2.12)  (n = 10) | 24.10  (19.40-35.00)  (n = 12) |
| Abbreviations: Hb = hemoglobin; LYMPH = lymphocytes; NEUT = neutrophils; PLT = platelets; RBC = red blood cells; WBC = white blood cells.  All parameters remained within their respective reference ranges, showing only minor fluctuations over time. The first row lists each parameter and its unit; the corresponding reference range is provided directly below. Data are presented as median with interquartile range, and *n* indicates the number of available values per parameter. | | | | | | | | |

*sTable 2B: Hematological Laboratory Values across Ocrelizumab Treatment Cycles*

| **Cycle** | **MONO/nL**  **0.20-0.60** | **MONO %**  **5.00-13.00** | **EOS/nL**  **0.06-0.46** | **EOS %**  **1.00-6.00** | **BASO/nL**  **<0.08** | **BASO %**  **<1.00** |
| --- | --- | --- | --- | --- | --- | --- |
| 0 | 0.51  (0.45-0.62)  (n = 54) | 8.20  (6.70-9.80)  (n = 97) | 0.13  (0.08-0.23)  (n = 55) | 2.00  (1.20-3.50)  (n = 97) | 0.04  (0.03-0.06)  (n = 46) | 0.80  (0.50-1.00)  (n = 94) |
| 1 | 0.56  (0.45-0.66)  (n = 58) | 8.90  (7.35-11.00)  (n = 93) | 0.12  (0.08-0.25)  (n = 56) | 2.00  (1.30-3.13)  (n = 94) | 0.04  (0.03-0.06)  (n = 46) | 0.80  (0.50-1.00)  (n = 90) |
| 2 | 0.56  (0.43-0.68)  (n = 50) | 9.10  (7.30-10.90)  (n = 89) | 0.14  (0.10-0.23)  (n = 47) | 2.35  (1.50-3.58)  (n = 86) | 0.04  (0.03-0.05)  (n = 43) | 0.70  (0.50-1.00)  (n = 85) |
| 3 | 0.58  (0.44-0.68)  (n = 44) | 9.50  (8.00-11.00)  (n = 67) | 0.14  (0.10-0.23)  (n = 44) | 2.40  (1.90-4.10)  (n = 67) | 0.04  (0.03-0.07)  (n = 39) | 0.90  (0.60-1.00)  (n = 62) |
| 4 | 0.62  (0.52-0.75)  (n = 49) | 9.10  (7.75-10.60)  (n = 61) | 0.20  (0.13-0.32)  (n = 48) | 3.00  (1.80-4.30)  (n = 59) | 0.05  (0.04-0.07)  (n = 44) | 0.90  (0.60-1.10)  (n = 60) |
| 5 | 0.61  (0.47-0.75)  (n = 42) | 9.55  (8.00-11.00)  (n = 54) | 0.13  (0.10-0.22)  (n = 41) | 2.20  (1.60-3.70)  (n = 52) | 0.04  (0.03-0.06)  (n = 34) | 0.70  (0.58-1.00)  (n = 50) |
| 6 | 0.60  (0.52-0.71)  (n = 37) | 9.20  (7.70-11.00)  (n = 43) | 0.15  (0.12-0.18)  (n = 38) | 2.30  (1.70-3.20)  (n = 43) | 0.05  (0.03-0.07)  (n = 34) | 1.00  (0.50-1.10)  (n = 43) |
| 7 | 0.63  (0.51-0.79)  (n = 23) | 10.10  (8.93-11.50)  (n = 32) | 0.17  (0.11-0.22)  (n = 26) | 2.30  (1.53-3.80)  (n = 32) | 0.05  (0.04-0.07)  (n = 23) | 0.80  (0.50-1.00)  (n = 31) |
| 8 | 0.67  (0.54-0.85)  (n = 23) | 9.70  (8.75-11.60)  (n = 25) | 0.21  (0.14-0.32)  (n = 23) | 3.00  (1.95-3.90)  (n = 25) | 0.05  (0.03-0.08)  (n = 20) | 0.70  (0.43-1.10)  (n = 24) |
| 9 | 0.59  (0.51-0.67)  (n = 10) | 8.50  (7.65-11.10)  (n = 12) | 0.13  (0.10-0.22)  (n = 9) | 2.10  (1.50-3.20)  (n = 11) | 0.06  (0.04-0.10)  (n = 9) | 1.00  (0.60-1.40)  (n = 11) |
| Abbreviations: BASO = basophils; EOS = eosinophils; MONO = monocytes.  All parameters remained within their respective reference ranges, showing only minor fluctuations over time. The first row lists each parameter and its unit; the corresponding reference range is provided directly below. Data are presented as median with interquartile range, and *n* indicates the number of available values per parameter. | | | | | | |

*sTable 3: IgG - Estimated Mean Differences vs. Baseline by Quartile*

|  | **Cycle** | **Mean Difference (mg/dL)** | **95% CI** | **p-value** |
| --- | --- | --- | --- | --- |
| **Quartile 1** | 1 | -17 | -105 to 71 | 0.99 |
|  | 2 | -47 | -110 to 17 | 0.26 |
|  | 3 | -57 | -170 to 55 | 0.61 |
|  | 4 | 18 | -103 to 140 | >0.99 |
|  | 5 | -51 | -103 to 140 | 0.56 |
|  | 6 | -35 | -162 to 91 | 0.97 |
|  | 7 | 8 | -116 to 132 | >0.99 |
|  | 8 | -11 | -211 to 189 | >0.99 |
|  | 9 | -31 | -1904 to 1841 | >0.99 |
| **Quartile 2** | 1 | -24 | -117 to 70 | 0.99 |
|  | 2 | -24 | -117 to 70 | 0.99 |
|  | 3 | 8 | -136 to 151 | >0.99 |
|  | 4 | -1 | -131 to 129 | >0.99 |
|  | 5 | -39 | -273 to 195 | >0.99 |
|  | 6 | 72 | -65 to 210 | 0.55 |
|  | 7 | 3 | -137 to 143 | >0.99 |
|  | 8 | 57 | -178 to 293 | 0.90 |
|  | 9 | 35 | -527 to 597 | 0.72 |
| **Quartile 3** | 1 | 6 | -64 to 77 | >0.99 |
|  | 2 | 48 | -35 to 132 | 0.55 |
|  | 3 | 57 | -34 to 148 | 0.43 |
|  | 4 | 80 | -42 to 202 | 0.36 |
|  | 5 | 27 | -68 to 122 | 0.97 |
|  | 6 | 5 | -93 to 102 | >0.99 |
|  | 7 | -39 | -483 to 405 | 0.98 |
|  | 8 | -26 | -353 to 302 | 0.99 |
|  | 9 | 12 | not available | not available |
| **Quartile 4** | 1 | 48 | -77 to 173 | 0.88 |
|  | 2 | 9 | -137 to 155 | >0.99 |
|  | 3 | 26 | -195 to 246 | >0.99 |
|  | 4 | -50 | -323 to 222 | >0.99 |
|  | 5 | 68 | -200 to 335 | 0.93 |
|  | 6 | 242 | -173 to 656 | 0.30 |
|  | 7 | 239 | -160 to 637 | 0.32 |
|  | 8 | 210 | -397 to 817 | 0.67 |
|  | 9 | 193 | -1253 to 1639 | 0.89 |
| Longitudinal changes in serum IgG stratified by baseline quartiles (n = 107). The mean rate of change per cycle was calculated by subtracting each subsequent value from the preceding one and averaging these differences across cycles. Statistical analysis was performed using a REML, and within-quartile changes were evaluated using Tukey’s multiple comparisons test (*p ≤ 0.05, **p ≤ 0.01, ***p ≤ 0.001). | | | | |

*sTable 4: IgM - Estimated Mean Differences vs. Baseline by Quartile*

|  | **Cycle** | **Mean Difference (mg/dL)** | **95% CI** | **p-value** |
| --- | --- | --- | --- | --- |
| **Quartile 1** | 1 | -10 | -18 to -2 | 0.01 |
|  | 2 | -14 | -20 to -2 | < 0.01 |
|  | 3 | -11 | -19 to -4 | < 0.01 |
|  | 4 | -13 | -22 to -4 | < 0.01 |
|  | 5 | -9 | -20 to -1 | 0.11 |
|  | 6 | -13 | -31 to -6 | 0.19 |
|  | 7 | -14 | -28 to -1 | 0.04 |
|  | 8 | -4 | -28 to -21 | 0.97 |
|  | 9 | -15 | -54 to -24 | 0.14 |
| **Quartile 2** | 1 | -17 | -31 to -4 | < 0.01 |
|  | 2 | -26 | -37 to -14 | < 0.01 |
|  | 3 | -26 | -39 to -12 | < 0.01 |
|  | 4 | -35 | -45 to -24 | < 0.01 |
|  | 5 | -32 | -44 to -20 | < 0.01 |
|  | 6 | -35 | -47 to -23 | < 0.01 |
|  | 7 | -39 | -55 to -24 | < 0.01 |
|  | 8 | -40 | -62 to -17 | < 0.01 |
|  | 9 | -46 | not available | not available |
| **Quartile 3** | 1 | -17 | -33 to 0 | 0.05 |
|  | 2 | -26 | -44 to -8 | < 0.01 |
|  | 3 | -35 | -55 to -17 | < 0.01 |
|  | 4 | -40 | -64 to -15 | < 0.01 |
|  | 5 | -40 | -61 to -19 | < 0.01 |
|  | 6 | -40 | -69 to -11 | 0.01 |
|  | 7 | -56 | -81 to -31 | < 0.01 |
|  | 8 | -34 | -138 to -72 | 0.36 |
|  | 9 | -27 | -434 to -380 | 0.68 |
| **Quartile 4** | 1 | -28 | -57 to -1 | 0.07 |
|  | 2 | -50 | -83 to -18 | < 0.01 |
|  | 3 | -59 | -96 to -22 | < 0.01 |
|  | 4 | -56 | -100 to -13 | < 0.01 |
|  | 5 | -85 | -124 to -45 | < 0.01 |
|  | 6 | -86 | -141 to -32 | < 0.01 |
|  | 7 | -89 | -171 to -8 | 0.03 |
|  | 8 | -87 | -164 to -10 | 0.03 |
|  | 9 | -77 | -157 to -3 | 0.05 |
| Longitudinal changes in serum IgM stratified by baseline quartiles (n = 107). The mean rate of change per cycle was calculated by subtracting each subsequent value from the preceding one and averaging these differences across cycles. Statistical analysis was performed using a REML, and within-quartile changes were evaluated using Tukey’s multiple comparisons test (*p ≤ 0.05, **p ≤ 0.01, ***p ≤ 0.001). | | | | |

*sTable 5: IgA: Estimated Mean Differences vs. Baseline by Quartile*

|  | **Cycle** | **Mean Difference (mg/dL)** | **95% CI** | **p-value** | |
| --- | --- | --- | --- | --- | --- |
| **Quartile 1** | 1 | 3 | -28 to 22 | >0.99 |  |
|  | 2 | -1 | -22 to 23 | >0.99 | |
|  | 3 | 1 | -25 to 22 | >0.99 | |
|  | 4 | -6 | -18 to 30 | 0.25 | |
|  | 5 | -3 | -20 to 27 | >0.99 | |
|  | 6 | -8 | -21 to 36 | >0.99 | |
|  | 7 | -7 | -32 to 45 | >0.99 | |
|  | 8 | -18 | -26 to 62 | 0.95 | |
|  | 9 | -5 | -67 to 77 | >0.99 | |
| **Quartile 2** | 1 | 7 | -29 to 14 | 0.99 | |
|  | 2 | -1 | -21 to 22 | >0.99 | |
|  | 3 | -3 | -21 to 26 | >0.99 | |
|  | 4 | -5 | -19 to 29 | >0.99 | |
|  | 5 | -7 | -19 to 33 | >0.99 | |
|  | 6 | -13 | -14 to 41 | 0.88 | |
|  | 7 | -16 | -45 to -16 | 0.77 | |
|  | 8 | -14 | -19 to 46 | 0.95 | |
|  | 9 | -25 | -64 to -13 | 0.54 | |
| **Quartile 3** | 1 | 4 | -28 to 20 | >0.99 | |
|  | 2 | -1 | -21 to 23 | >0.99 | |
|  | 3 | -8 | -15 to 31 | 0.98 | |
|  | 4 | -9 | -13 to 32 | 0.95 | |
|  | 5 | -13 | -37 to -12 | 0.82 | |
|  | 6 | -21 | -47 to -6 | 0.26 | |
|  | 7 | -27 | -62 to -9 | 0.34 | |
|  | 8 | -37 | -90 to -16 | 0.46 | |
|  | 9 | -51 | -125 to -24 | 0.48 | |
| **Quartile 4** | 1 | -17 | -43 to -8 | 0.50 | |
|  | 2 | -12 | -37 to -12 | 0.86 | |
|  | 3 | -14 | -43 to -14 | 0.86 | |
|  | 4 | -10 | -22 to 43 | >0.99 | |
|  | 5 | -42 | -68 to -17 | <0.01 | |
|  | 6 | -49 | -78 to -19 | <0.01 | |
|  | 7 | -60 | -88 to -32 | <0.01 | |
|  | 8 | -92 | -125 to -92 | <0.01 | |
|  | 9 | -93 | -167 to -20 | <0.01 | |
| Longitudinal changes in serum IgA stratified by baseline quartiles (n = 107). The mean rate of change per cycle was calculated by subtracting each subsequent value from the preceding one and averaging these differences across cycles. For Q1 IgA levels from cycle 9 were not available. Statistical analysis was performed using a REML, and within-quartile changes were evaluated using Tukey’s multiple comparisons test (*p ≤ 0.05, **p ≤ 0.01, ***p ≤ 0.001). | | | | | |

sTable 7: Univariable Analysis of the Association between LLN Immunoglobulin Levels and Infection Occurrence (COVID-19 cases included)

| **Variable** | **Patients with at least one documented infection**  **(n = 56)** | **Patients with**  **no documented infection**  **(n = 60)** | **p-value** | **Odds Ratio (95% CI)** |
| --- | --- | --- | --- | --- |
| LLN IgG at baseline | 3 | 9 | 0.14 | 0.35 (0.09-1.20) |
| LLN IgG during OCR | 9 | 9 | > 0.99 | 1.10 (0.41-2.80) |
| LLN IgM at baseline | 3 | 8 | 0.22 | 0.40 (0.11-1.40) |
| LLN IgM during OCR | 26 | 17 | 0.06 | 2.19 (1.04-4.57) |
| LLN IgA at baseline | 4 | 4 | > 0.99 | 1.20 (0.33-4.20) |
| LLN IgA during OCR | 5 | 4 | 0.74 | 1.40 (0.38-4.70) |
| Abbreviations: LLN = lower limit of normal; OCR = ocrelizumab.  Data were obtained from the study cohort (n = 116). Due to incomplete data, sample sizes varied for the following: immunoglobulin (IgG/IgM/IgA) at baseline n = 107. Univariable analyses were performed using Fisher’s exact test (*p ≤ 0.05, **p ≤ 0.01, ***p ≤ 0.001). | | | | |

*sTable 8: Univariable Analysis of Clinical and Treatment-Related Factors Associated with Herpes Zoster Occurrence*

| **Variable** | **Patients with Herpes Zoster**  **(n = 5)** | **Patients without**  **Herpes Zoster**  **(n = 111)** | **p-value** | **Odds Ratio (95% CI)** |
| --- | --- | --- | --- | --- |
| Age (≥ 40 years) | 2 | 61 | 0.66 | 0.55 (0.09-2.80) |
| Female | 3 | 67 | > 0.99 | 0.99 (0.19-5.70) |
| BMI ≥ 25 kg/m^2^ (n = 71) | 0 | 33 | 0.06 | 0.00 (0.00-0.82) |
| Any comorbidity | 4 | 49 | 0.18 | 5.10 (0.79-63.00) |
| RRMS | 5 | 89 | 0.58 | 0.00 (0.00-3.00) |
| ≥ 8 years of MS duration | 3 | 59 | > 0.99 | 1.30 (0.26-7.70) |
| Initial EDSS ≥ 3.5 (n = 105) | 1 | 56 | 0.36 | 0.31 (0.02-2.20) |
| ≥ 2 prior DMTs in pwRRMS (n = 94) | 4 | 63 | > 0.99 | 1.70 (0.26-22.0) |
| Prior interferon beta 1 a+b treatment (n = 94) | 1 | 54 | 0.17 | 0.19 (0.01-1.20) |
| Prior glatiramer acetate treatment (n = 94) | 1 | 40 | 0.39 | 0.31 (0.02-2.00) |
| Prior fingolimod treatment (n = 94) | 3 | 35 | 0.39 | 2.40 (0.46-14.00) |
| Prior natalizumab treatment (n = 94) | 2 | 36 | > 0.99 | 1.00 (0.17-5.10) |
| Prior dimethyl fumarate treatment (n = 94) | 1 | 27 | > 0.99 | 0.58 (0.05-3.80) |
| Prior high-dose corticosteroid treatment (n = 94) | 1 | 37 | 0.64 | 0.36 (0.03-2.30) |
| Prior plasmapheresis treatment (n = 94) | 0 | 5 | > 0.99 | 0.00 (0.00-18.00) |
| Persistent MRI-detected disease activity | 4 | 69 | 0.65 | 2.40 (0.38-30.00) |
| Hospitalization due to MS | 1 | 26 | > 0.99 | 0.82 (0.07-5.30) |
| Symptomatic medication | 2 | 72 | 0.35 | 0.36 (0.06-1.80) |
| ≥ 5 cycles of OCR | 3 | 66 | > 0.99 | 1.00 (0.20-5.90) |
| Lymphopenia | 2 | 25 | 0.33 | 2.30 (0.39-12.00) |
| Neutropenia | 0 | 3 | > 0.99 | 0.00 (0.00-28.00) |
| LLN IgG at baseline | 0 | 12 | > 0.99 | 0.00 (0.00-9.10) |
| LLN IgG during OCR | 0 | 18 | > 0.99 | 0.00 (0.00-3.90) |
| LLN IgM at baseline | 0 | 11 | > 0.99 | 0.00 (0.00-10.00) |
| LLN IgM during OCR | 3 | 40 | 0.36 | 2.70 (0.52-15.00) |
| LLN IgA at baseline | 0 | 8 | > 0.99 | 0.00 (0.00-15.00) |
| LLN IgA during OCR | 0 | 9 | > 0.99 | 0.00 (0.00-9.40) |
| Abbreviations: EDSS = Expanded Disability Severity Scale; LLN = lower limit of normal; DMT = disease-modifying therapy; MS = Multiple Sclerosis; OCR = ocrelizumab; RRMS = relapsing-remitting Multiple Sclerosis.  Data were obtained from the study cohort (n = 116). Univariable analyses were performed using Fisher’s exact test (*p ≤ 0.05, **p ≤ 0.01, ***p ≤ 0.001). Due to incomplete data, sample sizes varied for variables where specific n-values are indicated. | | | | |

***sFig. 1*** *Univariable Analysis of Clinical and Treatment-Related Factors Associated with Infection Occurrence*


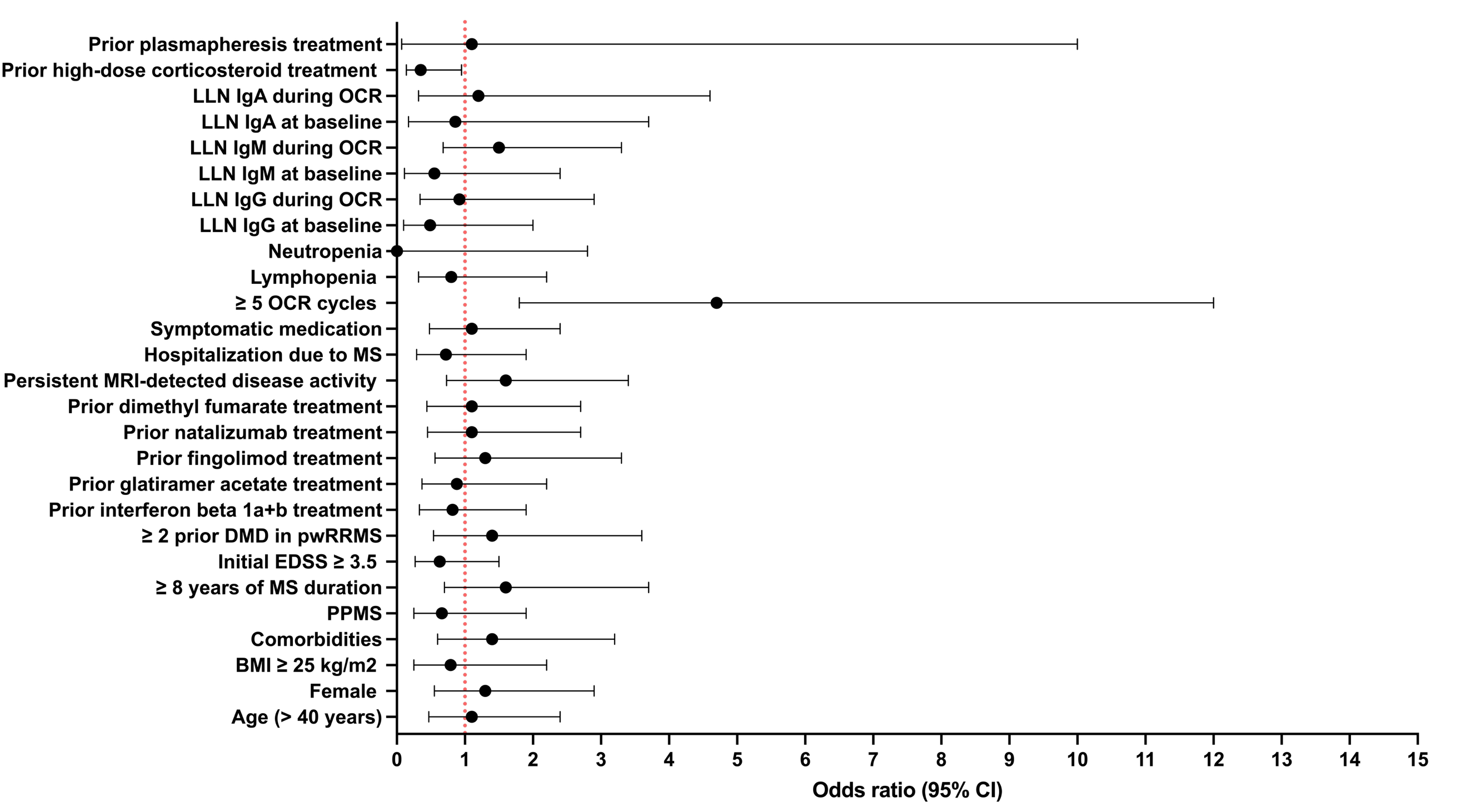


Univariable analysis evaluated clinical and treatment-related factors associated with infection occurrence in the study cohort (n = 116). Due to incomplete data, sample sizes varied for the following variables: BMI n = 71; EDSS n = 105; immunoglobulin (IgG/IgM/IgA) at baseline n = 107. Analyses of prior DMTs were performed exclusively in patients with relapsing-remitting MS (n = 94). Each point represents the odds ratio (OR) with 95% confidence intervals. ORs >1 indicate increased odds of experiencing an infection based on clinical history. Parameters with 95% CIs not crossing 1 were considered statistically significant (α = 0.05). Univariable analyses were conducted using Fisher’s exact test. DMT = disease-modifying therapy; EDSS = Expanded Disability Severity Scale; LLN = lower limit of normal; MS = Multiple Sclerosis; OCR = ocrelizumab; PPMS = primary progressive multiple sclerosis; pwRRMS = patients with relapsing-remitting MS.

***sFig. 2*** *Univariable Analysis of Clinical and Treatment-Related Factors Associated with Herpes Zoster*


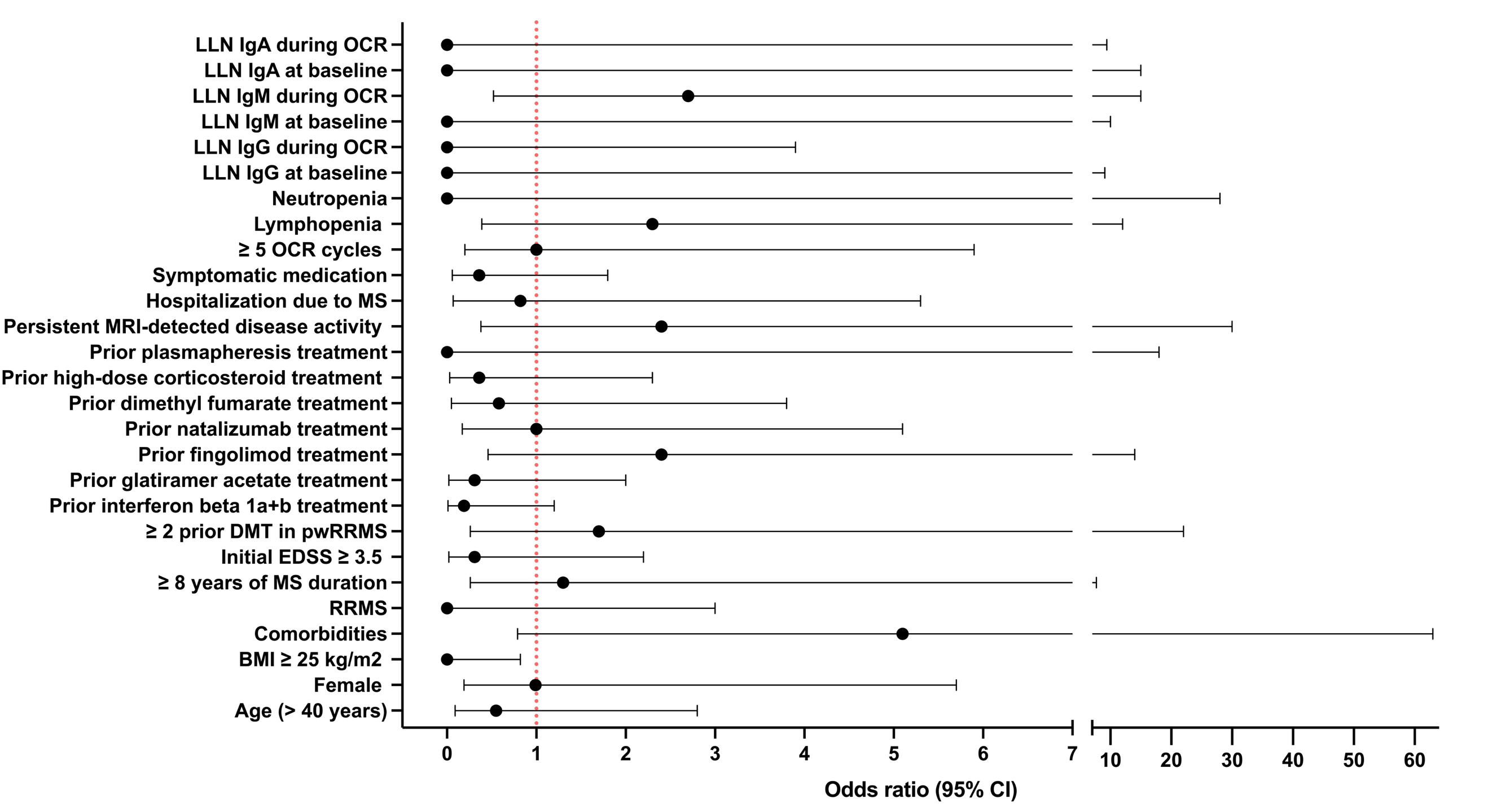


Data were derived from the study cohort (n = 116), including participants with herpes zoster (n = 5) and without herpes zoster (n = 111). Due to incomplete data, sample sizes varied for the following variables: BMI n = 71; EDSS n = 105; immunoglobulin (IgG/IgM/IgA) at baseline n = 107. Analyses of prior DMTs were performed exclusively in patients with relapsing-remitting Multiple Sclerosis (n = 94). Each point represents an odds ratio, with horizontal lines indicating the 95% confidence intervals. Odds ratios > 1 indicate an increased likelihood of herpes zoster occurrence. Univariable associations were assessed using Fisher’s exact test. DMT = disease-modifying therapy; EDSS = Expanded Disability Severity Scale; Ig = immunoglobulin; LLN = lower limit of normal; MS = Multiple Sclerosis; OCR = ocrelizumab; pwRRMS = patients with relapsing-remitting MS; RRMS = relapsing-remitting Multiple Sclerosis.

***sFig. 3*** *Longitudinal Anti-VZV Immunoglobulin Responses During Ocrelizumab Therapy*


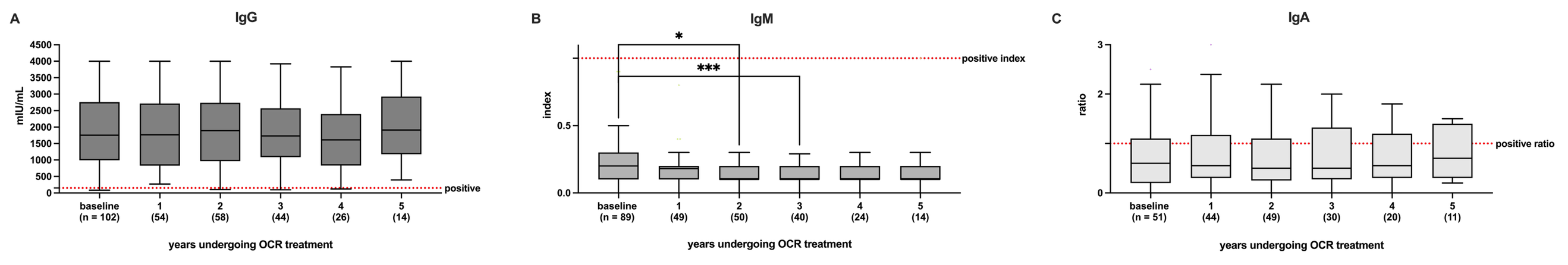


The x-axis displays baseline measurements and annual follow-up during OCR therapy. The y-axes represent (A) anti-VZV IgG (mIU/mL), (B) anti-VZV IgM (index), and (C) anti-VZV IgA (ratio). Data are shown as median and IQR using box-and-whisker plots. Boxes indicate the IQR (25^th^–75^th^ percentile), horizontal lines show the median, and whiskers extend to 1.5 × IQR. *n* denotes the number of available measurements per time point. Baseline VZV seropositivity was documented for all patients; however, quantitative baseline VZV antibody values were not available in all cases, resulting in varying sample sizes at baseline. Statistical analysis was performed using a REML model with Tukey’s multiple comparisons test. p values were set at *p ≤ 0.05, **p ≤ 0.01, and ***p ≤ 0.001. Ig = immunoglobulin; OCR = ocrelizumab.
